# The Status of Infectious Disease in the Amazon Region

**DOI:** 10.3201/eid1504.090169

**Published:** 2009-04

**Authors:** Pedro Luiz Tauil

**Affiliations:** University of Brasília, Brasília, Brazil

**Keywords:** Infectious disease, Amazon, Brazil, malaria, commentary

The Amazon River basin region is a vast territory with an area >7 million km^2^, encompassing parts of 9 South American countries: Bolivia, Brazil, Colombia, Ecuador, French Guiana, Guyana, Peru, Suriname, and Venezuela. The Amazon River, the longest river in the world, traverses the region from west to east, fed by multiple tributaries. The region also contains the largest tropical rainforest in the world, situated on a massive plain whose altitude is near sea level. With a climate characterized by high temperatures and humidity and copious rainfall, the region has the densest and most varied ecosystem in the world.

Conditions in the region are favorable for the transmission of numerous tropical diseases, which pose particular risks for populations exposed to precarious housing and working conditions. Many of these are well-known diseases whose epidemiologic characteristics are changing as the result of accelerating population, environmental, and climate changes. Others are novel diseases, which are being discovered in the region regularly.

Malaria is the most important endemic disease in the region because of its high incidence. It is naturally transmitted by mosquitoes of the genus *Anopheles.* Slow-flowing, nonpolluted, shaded waters in the region provide this vector with a favorable environment for reproduction, and dense forest enables the adult vector to live longer than in other climates. Climatic conditions favor the development of *Plasmodium* spp. in these mosquitoes. Intense human migration from rural to urban areas contributes to malaria transmission in peripheral areas of Amazonian cities.

Tegumentary leishmaniasis is another high-incidence disease in the region. Multiple animal species serve as reservoirs for *Leishmania* spp. in the rainforest, as do the disease’s primary vectors, insects of the genus *Lutzomya*. Arboviruses are highly endemic to the region, particularly Oropouche virus and Venezuelan equine encephalitis virus. Yellow fever is also endemic; nonhuman primates are the principal reservoirs of the disease during its sylvatic cycle. Vaccination is an essential means of protection against yellow fever for both the local population and visitors. *Aedes aegypti* mosquitoes in Amazonian urban centers pose an ever-present risk for yellow fever transmission and are also responsible for the high incidence of dengue.

For reasons yet to be determined, persons living in the Amazon region have a high prevalence of leprosy and viral hepatitis. Mycoses such as Jorge Lobo disease, caused by *Blastomyces loboi*, are also characteristic of the region (*1*).

The transmission of known pathogens through novel modes, the emergence of pathogens not previously detected in the region, and the emergence of newly recognized pathogens have been reported with increasing frequency in the Amazon region. As illustrated by articles in this issue about adiaspiromycosis (*2*), malaria (*3*,*4*), dengue (*4*), Chagas disease (*5*), Kaposi sarcoma–associated herpesvirus (*6*), suspected Brazilian purpuric fever (*7*), and other infections, new diseases continue to emerge and old ones continue to undergo epidemiologic change throughout the vast Amazon River basin.
